# Systematic and Iterative Development of a Smartphone App to Promote Sun-Protection Among Holidaymakers: Design of a Prototype and Results of Usability and Acceptability Testing

**DOI:** 10.2196/resprot.7172

**Published:** 2017-06-12

**Authors:** Angela M Rodrigues, Falko F Sniehotta, Mark A Birch-Machin, Patrick Olivier, Vera Araújo-Soares

**Affiliations:** ^1^ Institute of Health and Society Newcastle University Newcastle United Kingdom; ^2^ Institute of Cellular Medicine Newcastle University Newcastle Upon Tyne United Kingdom; ^3^ Open Lab School of Computing Science Newcastle University Newcastle Upon Tyne United Kingdom; ^4^ Institute of Health & Society Newcastle University Newcastle Upon Tyne United Kingdom

**Keywords:** sun-protection, sunburn, sunscreening agents, sunbathing, health behavior, health promotion, formative research, intervention

## Abstract

**Background:**

Sunburn and intermittent exposure to ultraviolet rays are risk factors for melanoma. Sunburn is a common experience during holidays, making tourism settings of particular interest for skin cancer prevention. Holidaymakers are a volatile populations found at different locations, which may make them difficult to reach. Given the widespread use of smartphones, evidence suggests that this might be a novel, convenient, scalable, and feasible way of reaching the target population.

**Objective:**

The main objective of this study was to describe and appraise the process of systematically developing a smartphone intervention (mISkin app) to promote sun-protection during holidays.

**Methods:**

The iterative development process of the mISkin app was conducted over four sequential stages: (1) identify evidence on the most effective behavior change techniques (BCTs) used (active ingredients) as well as theoretical predictors and theories, (2) evidence-based intervention design, (3) co-design with users of the mISkin app prototype, and (4) refinement of the app. Each stage provided key findings that were subsequently used to inform the design of the mISkin app.

**Results:**

The sequential approach to development integrates different strands of evidence to inform the design of an evidence-based intervention. A systematic review on previously tested interventions to promote sun-protection provided cues and constraints for the design of this intervention. The development and design of the mISkin app also incorporated other sources of information, such as other literature reviews and experts’ consultations. The developed prototype of the mISkin app was evaluated by engaging potential holidaymakers in the refinement and further development of the mISkin app through usability (ease-of-use) and acceptability testing of the intervention prototype. All 17 participants were satisfied with the mISkin prototype and expressed willingness to use it. Feedback on the app was integrated in the optimization process of the mISkin app.

**Conclusions:**

The mISkin app was designed to promote sun-protection among holidaymakers and was based on current evidence, experts’ knowledge and experience, and user involvement. Based on user feedback, the app has been refined and a fully functional version is ready for formal testing in a feasibility pilot study.

## Introduction

Skin cancer incidence within white populations has been increasing worldwide [1-3]. Exposure to ultraviolet radiation (UV) and history of sunburn—both modifiable behavioral factors—are considered the major etiologic factors for melanoma [4-6]. Epidemiologic studies suggest that sun-safe behaviors, such as wearing protective clothes, avoiding sun-exposure at midday, and sunscreen use would decrease the amount of intermittent sun-exposure and have an important effect in reducing skin cancer incidence [7].

Skin cancer is the most common form of all types of cancer diagnosed in the United Kingdom [8]. The age-standardized melanoma incidence rate for 2010 was 17.1 per 100,000 people in the population. In the same year, malignant melanoma was the fifth most common type of cancer [9]. The number of individuals engaging in risk behaviors during their holidays is increasing. Sunburn is a common experience while on holiday [10,11] and sun-related behaviors, such as intentional sun-seeking behavior, are increasingly high [12,13]. In the United Kingdom, studies evaluating effectiveness of sun-protection interventions in recreational settings are sparse. Currently, the SunSmart campaign implemented by the Cancer Research, UK [14], is the major intervention being rolled out.

Considering the time of day and location barriers in interventions designed to target holidaymakers, it seems easy to conclude that interventions that use mobile computing and communication technologies (eg, smartphones and personal digital assistants) are potentially an effective option for skin cancer prevention. Several systematic reviews have demonstrated the potential of mobile technologies to change health-related behavior [15-18]. Text messaging services have been shown to be a valuable strategy in increasing sunscreen use [19] and, more generally, sun-safety behaviors in young Australian adults [20].

One of the main problems identified in a previous systematic review of interventions delivered in tourism settings was the lack of detail on the development process [21]. The Medical Research Council guidance on the development of complex interventions is widely recognized and entails a specific set of processes and methods that will enable replication and transparency [2,3]. The first step, and the target of this paper, is to synthetize the best available and most appropriate evidence that can then be used to inform the specific features of the intervention. A key element in the development of new technologies for behavior change is whether it suits its purpose and meets users’ needs and expectations [22]. This information can help intervention developers understand user perspectives on the topic and intervention features, which can maximize the acceptability of the newly developed intervention [23].

A recent study [24] designed and developed a mobile-phone app to promote sun-protection, using a user-centered design. Four rounds of usability testing were implemented by conducting focus groups with 22 potential users. Participants rated the Solar Cell app favorably and described it as being “user friendly.” The process of intervention development in this app did not report the use of evidence from recent systematic reviews in the area of skin cancer prevention [21,25,26]. For instance, the intervention could have benefited from using some of the strategies suggested, such as stimulating social norms and providing appearance-based information about photoaging with UV photographs [21].

As identified by a recent systematic review [21], previous studies conducted in the skin cancer prevention area had several shortcomings: (1) measurement procedures (eg, lack of objective measures), (2) study design (eg, mainly uncontrolled before-after), (3) poor intervention description and reporting, (4) lack of systematic development building on established knowledge, and (5) poor description of theory base. One of the gaps in this review was related to the fact that no mobile-phone interventions had been available or tested when the review was completed. According to the Medical Research Council framework [2,3], systematic reviews with behavior change techniques (BCTs) analysis provide a starting point for intervention development. However, there are still uncertainties on how to (1) fill the gaps where the evidence base is limited, (2) establish theory, and (3) engage experts from different fields in the development process.

This paper provides an innovative approach on how to integrate different sources of information in a thorough, systematic, and iterative development process for the *mISkin* app, targeting sun protection. By consistently describing how behavioral interventions are derived and decisions made based on specific constraints, more transparency and reproducibility will be achieved in the area of skin cancer prevention. The main objective of this study is to describe and appraise the process of systematically developing a smartphone based intervention (*mISkin* app) to promote sun-protection during holidays. This process incorporates both theory and evidence-based approaches outlined by the Medical Research Council framework [2,3], engaging users perspectives in the development process of the *mISkin* app [22,23].The specific research questions this paper will answer are as follows: (1) What are the different strands of evidence used to inform the development of the mock-up prototype of the smartphone app? (2) What are the participants’ routines during holidays and how can a sun-protection app fit into these routines? and (3) What are the potential app users’ reactions to or interactions with a mock-up prototype?

## Methods

### Ethics

The study was approved by the Faculty of Medical Sciences Ethics Committee (Newcastle University) (Reference no: 00427_2/2013).

### Overview

**Figure 1:**
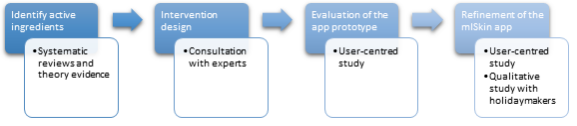
Overview of the stages of the development process for the mISkin intervention.

The development process of the *mISkin* app was conducted over four sequential stages (Figure 1): (1) identify evidence on the most effective behavior change techniques (BCTs) used (active ingredients) as well as theoretical predictors and theories, (2) evidence-based intervention design, (3) co-design with users of the *mISkin* app prototype, and (4) refinement of the app. Each stage provided key findings that were subsequently used to inform the design of the *mISkin* app. Figure 1 provides an overview of the sequential stages of the development process. This section presents the procedures and key findings used to inform the next stage of the iterative development process. A brief summary of the rationale for the development of a smartphone intervention to promote sun-protection during holidays is also provided.

### Stage 1: Identifying Active Ingredients, Behavior Change Theory, and Modes of Delivery Evidence

#### Procedure

Evidence from a systematic review assessing the efficacy of interventions promoting sun-protection behaviors in holiday settings [21] conducted by the authors and wider evidence for behavior change was synthesized by the research team.

The content of the interventions was coded using a taxonomy of behavior change techniques [27]. Methods of delivery were coded in terms of the format (ie, individual or group/community), content (ie, verbal communication, written material, videos, photographs, interactive activities, and environmental resources), provider (ie, professionals delivering the intervention materials), and setting (ie, location) of the intervention [28].

#### Analysis

Data from the relevant literature was extracted and collated in a mapping exercise that covered included BCTs and rationale for inclusion. The list of included BCTs and rationale was used to inform the content of the intervention in stage 2.

### Stage 2: Consultations With Experts and Intervention Design

#### Procedure

After gathering, collecting, and analyzing information regarding state-of-the-art evidence, the design and concept development of the smartphone app was informed and overseen by an interdisciplinary group of experts in dermatology, behavior change, and computing science. Experts presented experience in designing, developing, and evaluating theory-based interventions and psychological theories of behavioral change, experience on the cutaneous response to ultraviolet radiation (UVR), and development and application of pervasive computing interactive technologies for health and well-being.

Two main sets of expert consultations were conducted: (1) behavioral scientists plus computer scientists and (2) behavioral scientists plus dermatological science researchers and vitamin D experts. The meetings were frequent during the early stages of app design and initial testing (ie, approximately every month). These were structured by defining clear agenda items beforehand and by keeping detailed minutes of main decisions discussed. After reaching agreement over the overarching themes and design, the meetings with app developers were regular to evaluate the progress of the app design.

#### Analysis

Translating the interventions content into app features involved repeated iterations between members of the research team conducting the systematic review of the current available evidence and the members of the team with expertise on app development. The initial step was to agree on the theoretical basis and principles underpinning the intervention based on previous studies. This evidence informed the production of guiding principles that described the key features to be included in the app. These discussions also explored the best ways to deliver the BCTs selected and the agreed guiding principles for the intervention. After reaching an agreement over the key app features and design, we met regularly to evaluate progress of the app design.

Data retrieved from these discussions were collated to develop interactive mock-ups and a workflow for the *mISkin* app.

### Stage 3: Evaluation of the mISkin App Prototype

#### Participants

A total of 17 adults (13 females) aged 21-62 years (mean=36.8, SD=11.3) participated in the semistructured interviews. Participants were recruited through advertisement leaflets placed across Newcastle upon Tyne, United Kingdom.

#### Procedure

The aim of this stage was to obtain an exploratory evaluation of the intervention principles and resulting prototype. Individual interviews were used to obtain feedback and suggestions for improvement by giving the participants a key role in optimizing the intervention (co-design).

Eligible participants had to be over 18 years old with past experience of sunny holidays abroad. Included participants were assessed for inclusion criterion and were required to provide informed consent before participation. Interviews were conducted by a female researcher (AR) with experience in interviewing. Interviews lasted between 30-50 minutes and were audio-recorded and transcribed.

**Figure 2:**
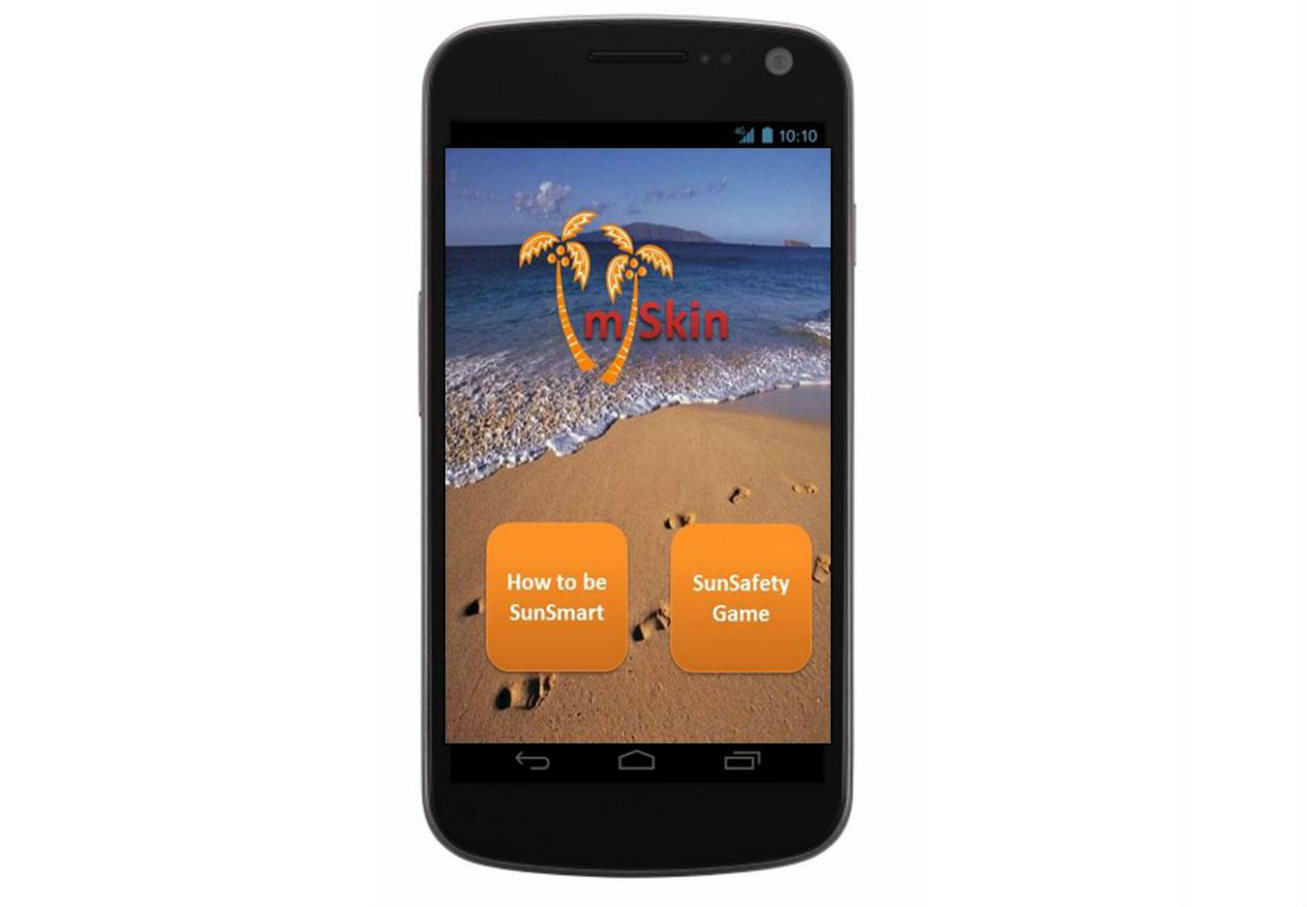
The mISkin main screen mock-up.

Participants were shown the interactive mock-up of the *mISkin* app (Figure 2), with a brief demonstration of the main functionalities of the app. Participants were asked to interact with the mock-up and provide feedback, highlighting their satisfaction and dissatisfaction with the design, content, and format. Individuals were asked to provide suggestions for improvement (Multimedia Appendix 1: [Co-design individual interview topic guide]).

#### Analysis

All transcripts were imported into NVivo 10.0. Participants’ feedback on the prototype was summarized into main suggestions, in order to refine the smartphone app before the feasibility pilot study.

### Stage 4: Refinement of the mISkin App

#### Procedure

Based on the engagement and experience of the participants, a list of suggestions for improvement was compiled for discussion within the research team. In the participants’ views, these suggestions would improve the acceptability and usability of the *mISkin* app. The way these were prioritized and implemented was based on whether their feedback indicated that a feature was potentially off-putting.

This stage was also informed by the key findings from a qualitative study with holidaymakers reported elsewhere [30].

#### Analysis

There were some discrepancies between the data gathered in this stage and the guiding principles produced by the research team in an earlier stage. The feedback provided by participants on the UV photographs was mixed (ie, both positive and negative reactions). However, visualizing UV photographs was an essential component of our intervention package, as previous studies had shown that these images might be effective in motivating people to improve sun-protection practices whilst on holiday [21]. In addition, findings from the qualitative study [30] suggested that respondents showed a desire to tan and attributed a high value to a tanned appearance during holidays. The conclusions also suggest that future public health messages should highlight the harmful effects of sunlight on appearance and demonstrate effective ways of performing sun-protection practices (eg, applying sunscreen properly).

Despite some users expressing negative reactions to the UV photographs (eg, fear or worry), all participants thought it was an important element to be included in the app. Consequently, UV photographs were retained in the *mISkin* app, but risk communication was improved in order to provide more health-related information before showing the UV pictures to the users.

Another discrepancy was the assumption about the potential disruptiveness of the Sun Alert service for users. The initial mock-up only assumed two prompts per day in order to avoid annoying users with alerts. However, feedback given by users was to make this feature customizable as some would have liked to receive more than two prompts per day.

## Results

### Stage 1: Identifying Active Ingredients, Behavior Change Theory, and Modes of Delivery Evidence

#### Findings

Table 1 details the list of included BCTs and presents the rationale. A systematic review of previously tested interventions to promote sun-protection provided pointers and evidence-based constraints for the design of this intervention, allowing for evidenced-based intervention development [21]. The pointers comprised effective BCTs and the need for any intervention targeting holidaymakers to be scalable and geographically flexible, delivered “on site” and not before holidays. The evidence-based constraints that had to be dealt with were as follows: (1) the lack of details on the development process of previous interventions, (2) the lack of mobile-phone interventions identified in the systematic review conducted, and 3) the lack of evidence available in the systematic review on the “how to” procedures for the intervention delivery.

The analysis of the active components provided useful information for intervention development [21]. We followed the recommendations of the systematic review to include BCTs that were consistently present in all interventions previously conducted in the field, by providing information on the consequences of performing sun-protection and on how to perform relevant sun-safety behaviors. The BCTs most strongly associated with effective interventions were also included in the app, namely (1) stimulate supportive social norms for sun-protection behaviors (eg, providing information about others’ behavior and social norms), and (2) provide appearance-based information about skin photoaging, illustrated with UV photographs of skin damage. While the findings of the systematic review are informative, they are not considered to be definitive or sufficient to fully design a behavioral intervention and, therefore, other information sources were used to inform the development of all the components of the *mISkin* app, such as other systematic reviews and consultations with experts (eg, the need to include information on Vitamin D). Evidence on the most effective BCTs and associated theoretical mediators from other systematic reviews targeting behavior change [15,25,26,31,32] was also used to inform this stage.

This review [21] also found evidence for different modes of delivery. Interventions using written information and face-to-face communication were more effective than interventions using interactive sessions. Interactive activities were defined as demonstrations, games, and puzzles, used primarily with children to promote sun-protection. Delivering the intervention on site (eg, a holiday resort) was more effective than delivering pre-exposure.

Even though the systematic review did not provide specific evidence regarding smartphone use as a possible mode of delivery, other evidence suggested that this might be a novel, convenient, scalable, and feasible way of reaching the target population [15-18], given the widespread use of smartphones. Smartphones hold several advantages for behavioral medicine: (1) embedded location information (eg, GPS) can provide many important opportunities for hard to reach populations, (2) continuous uninterrupted data log, (3) capacity to support various multimedia apps, and (4) portability [29]. Holidaymakers are a volatile population present at different locations, which may make them difficult to reach. A scalable and geographically flexible smartphone intervention might be an effective way of reaching this population.

### Stage 2: Consultations With Experts and Intervention Design

#### Findings

The intervention principles guided the design and development process of the app and added value to the experts’ consultations. The key principles introduced based on expert advice were as follows: (1) development of an algorithm for sun-protection reminders (ie, using smartphone GPS data on indoor or outdoor locations), (2) vitamin D advice, and (3) introduction of gamification to deliver sun-protection information.

Any discrepancies between the different sources of data were solved based on the pre-specified intervention principles. For instance, the Vitamin D topic was added after consultation with experts in this area. However, this addition was congruent with our guiding principles in terms of providing health-related information within the app. Consultations with experts were essential to further develop and refine the intervention based on the iterative discussions with the core team members and wider consultations. One of the key challenges was the need to adapt to similar technical language when communicating within a multidisciplinary team.

**Figure 3:**
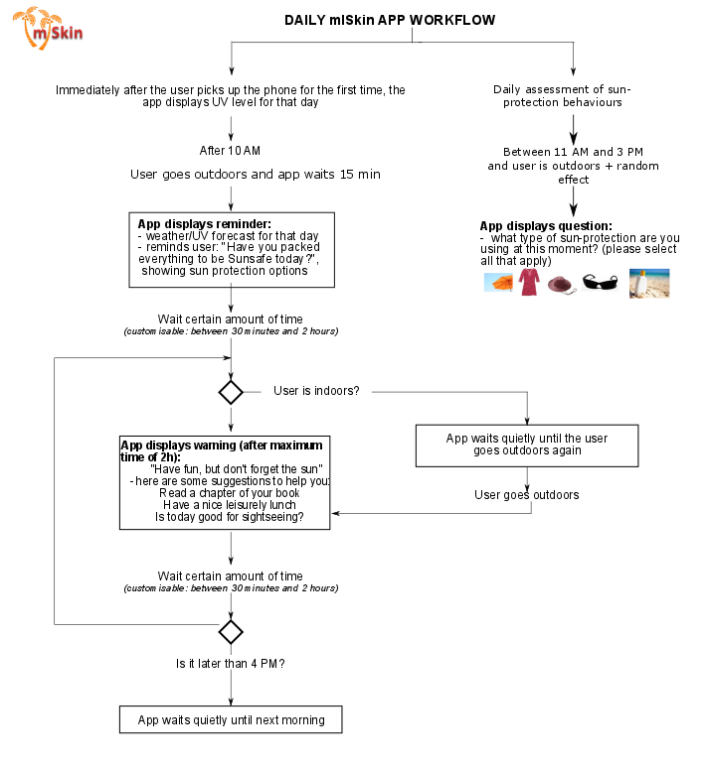
The mISkin app workflow.

The developed interactive mock-up and workflow of the *mISkin* app are presented in Figure 2 and Figure 3 respectively. Interactive mock-ups of the app were developed and used to test for ease-of-use, graphics appeal, and the general comprehension and acceptability of the distinct features of the *mISkin* app, using semi-structured interview methods.

The workflow was used as a design brief depicting the interaction process within the *mISkin* app that informed the development of a functional version of the intervention prototype by the app developers.

**Table 1:** Description of the *mISkin* app’s main features, including behavior change techniques and rational for inclusion.

Feature	Description	Behavior change techniques[27]	Rational for inclusion (evidence-based and theory-based)
Skin sensitivity assessment with feedback	A set of 5 questions about skin reaction to the sun, based on previous literature (eg, [33,34]. After completion, participants receive feedback about their specific skin type and their reaction to the sun (eg, “You have skin type III. Sometimes burns, usually tans”).	Provide information on consequences of behavior to the individual.	Understanding their personal risk of sunburn will help people shape outcome expectations, which in turn will impact goal setting. Evidence: A systematic review [21] outlines the importance of understanding the consequences of excessive sun-exposure. Theory: Social Cognitive Theory postulates that people tend to form outcome expectancies about the results of given actions [35]. In line with these outcome expectancies, people will engage in actions that are likely to produce positive outcomes and dismiss those that result in negative consequences [35].
NHS^a^ Choices “How to apply sunscreen” Video^b^	The video provides information how to properly apply sunscreen, stating specific information about quantity, frequency, SPF, how to apply it before leaving the house, where to apply it, and guidance on sunscreen costs. The video also demonstrates how to apply sunscreen properly by showing a model doing it. The importance of other methods of sun-protection is also discussed in the video (ie, covering up and seeking shade). Special attention is devoted to children and the need for additional information about sun-protection. The risk of sunburn and skin cancer is also highlighted in the video. A snapshot from the NHS Choices video “How to be Sun Smart” was also included to foster social comparison on sun-protection habits.	Provide information on consequences of behavior in general, provide information on where and when to perform the behavior, provide instructions on how to perform the behavior, and demonstrate the behavior.	The video tackles all important instructions regarding sunscreen app, providing a complete display of the “how to do it” technique. The video also provides information about other methods of sun-protection and the consequences of excessive sun-exposure. Evidence: systematic review [21]. Theory: In the Social Cognitive Theory, instructions on how to engage in a specific behavior are essential to translate a goal into action, which will in turn foster self-efficacy and subsequent further behavior change [36].
UV^c^ photographs	The app submenu “How to be SunSmart” also includes ultraviolet photographs of the face (male and female). Before displaying the pictures, a brief description is provided.	Provide information on consequences of behavior in general; fear appeals.	The inclusion of these types of photographs helps highlight the harmful effects of exposure to ultraviolet rays on people’s appearance and, subsequently, promotes sun-protection habits. Evidence: Various systematic reviews [21,25,26]. The desire to have a tan is a central motive for sun exposure, as most people believe that a tan will improve personal appearance (eg, [37-39]. Research also shows that people find others more attractive when they have a tan [21,37,38]. Thus, interventions that highlight the negative effects of exposure to ultraviolet rays on one’s appearance might lead to significant behavior change (eg, [21,37]. Theory: Social Cognitive Theory hypothesizes that people will engage in actions that are likely to produce positive outcomes based on outcome expectancies [35].
“Sun safety quiz”	This component engages holidaymakers in the “Sun Safety Quiz” by answering true or false to questions on general principles of sun-protection practices, information on positive consequences of sun-protection, tanning, vitamin D and UV Index. This is a gamification component, in which participants receive performance-based rewards (ie, positive feedback and final score message). Feedback provided also highlights others’ use of sun-protection to facilitate social comparison.	Provide feedback on performance, provide information on consequences of behavior in general, provide information about others’ approval, provide normative information about others’ behavior, and facilitate social comparison.	A gamification feature was included in the quiz with feedback about performance and the provision of relevant information to facilitate social comparison. Evidence: Even though no conclusive evidence was unveiled by the completed systematic review [21], other systematic reviews have shown that self-regulatory strategies [31,40] and gamification [21,41,42] can be effective in changing other health behaviors. Theory: According to the Control Theory [43], feedback on performance provides external feedback on achievements and can lead to behavioral change. The Social Cognitive Theory hypothesizes that referential performance is induced by a process of social self-judgment, where social comparison is central. The provision of opportunities for social comparison is therefore an important strategy to influence referential performances and promote behavior change [35].
“Sun Alert service”	An algorithm was designed to define the main rules for interaction between the app and participants (Figure 3). This interaction is especially important to establish rules for delivering prompts for sun-protection. These prompts will occur between 10 AM and 4 PM and will depend on participant location (indoors or outdoors information based on smartphone GPS). Participants will receive approximately 2 prompts per day. In these prompts, a forecast of the levels of ultraviolet radiation will also be provided.	Prompt practice	Several studies showed that forgetfulness is a key barrier for sun-protection [44]. We believe that prompting will help individuals remember about sun-protection methods at least at two points in the day: (1) the start of the day, just before temperature starts increasing (ie, 10 AM), and (2) at midday when sun-protection (eg, seeking shade) is most needed. Evidence: Systematic reviews [15,21,31] and a previous trial on sunscreen use [19]. Theory: The Social Cognitive Theory envisages prompting as a key strategy for behavior change. Prompting enables individuals to experience mastery which promotes self-efficacy [45].
Diary record: ecological momentary assessment	Real-time data capture through the smartphone app is also used for assessment of sun-protection practices. This assessment will occur randomly between 11 AM and 3 PM, if the individual is outside (as detected by the GPS on the smartphone). Sun-protection practices will be represented by the use of symbols or pictures (please see Figure 5) and participants will only need to touch the screen to record the use of sun-protection.	Prompt self-monitoring	Self-report is prone to inaccuracies and biases in the reporting of behavior [46]. Smartphones can be an effective and feasible alternative to self-report for sun-protection assessment, especially because these devices can collect behavioral events in natural settings and produce time- and date- stamp events [47]. Evidence: Previous systematic reviews have shown the efficacy of this strategy in changing behavior [31,32,47,48]. Theory: Self-monitoring is a key strategy for behavior change for both the Control theory [43] and the Social Cognitive Theory [36]. Monitoring present behavior can lead to comparisons between actual behavior and standards and, subsequently, adjustments in performance in order to reach behavioral standards.

^a^NHS: National Health Service.

^b^Permission was granted by NHS Choices to be used in the mISkin application.

^c^UV: Ultraviolet.

**Figure 4:**
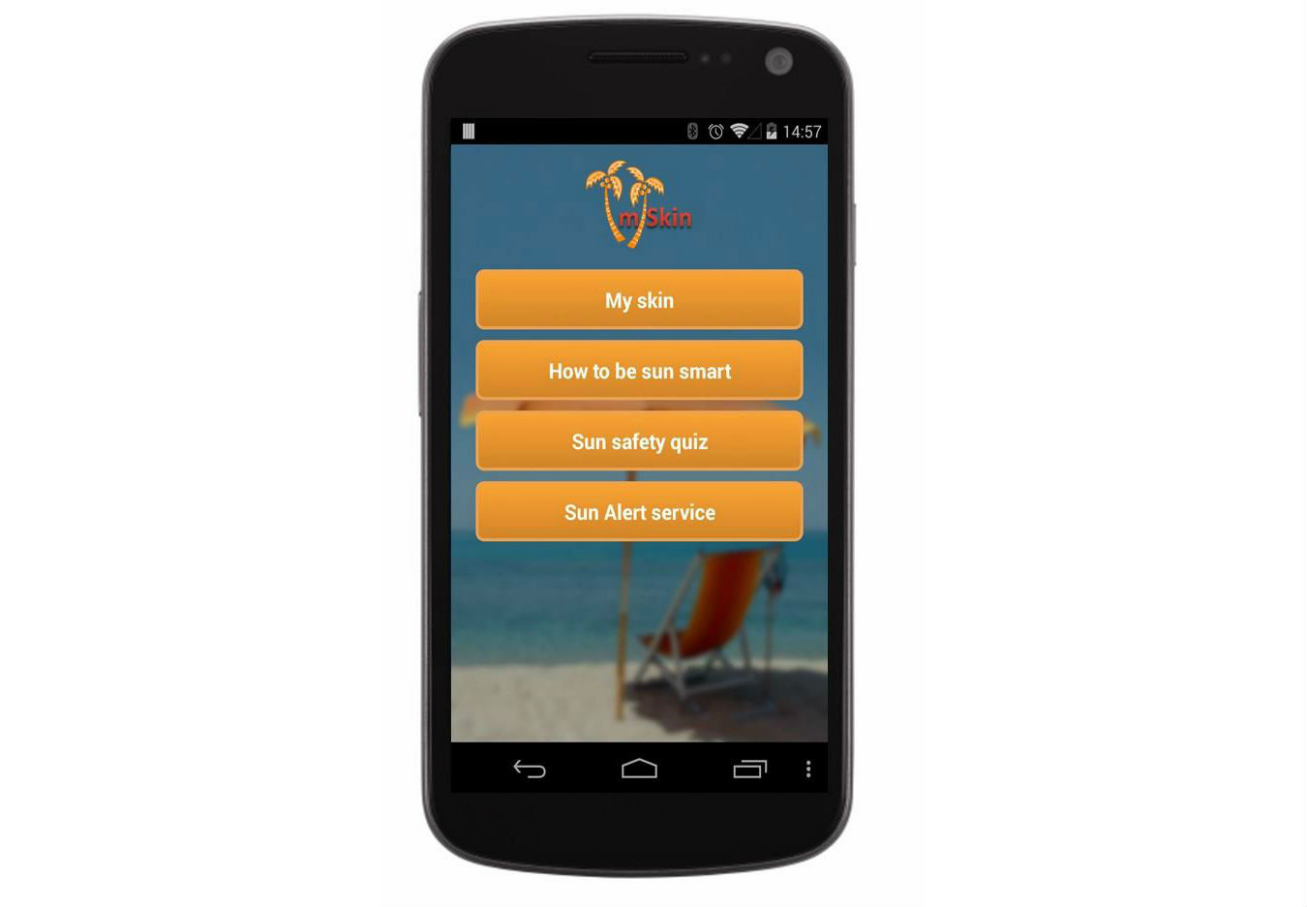
Main screen of the mISkin app.

**Figure 5:**
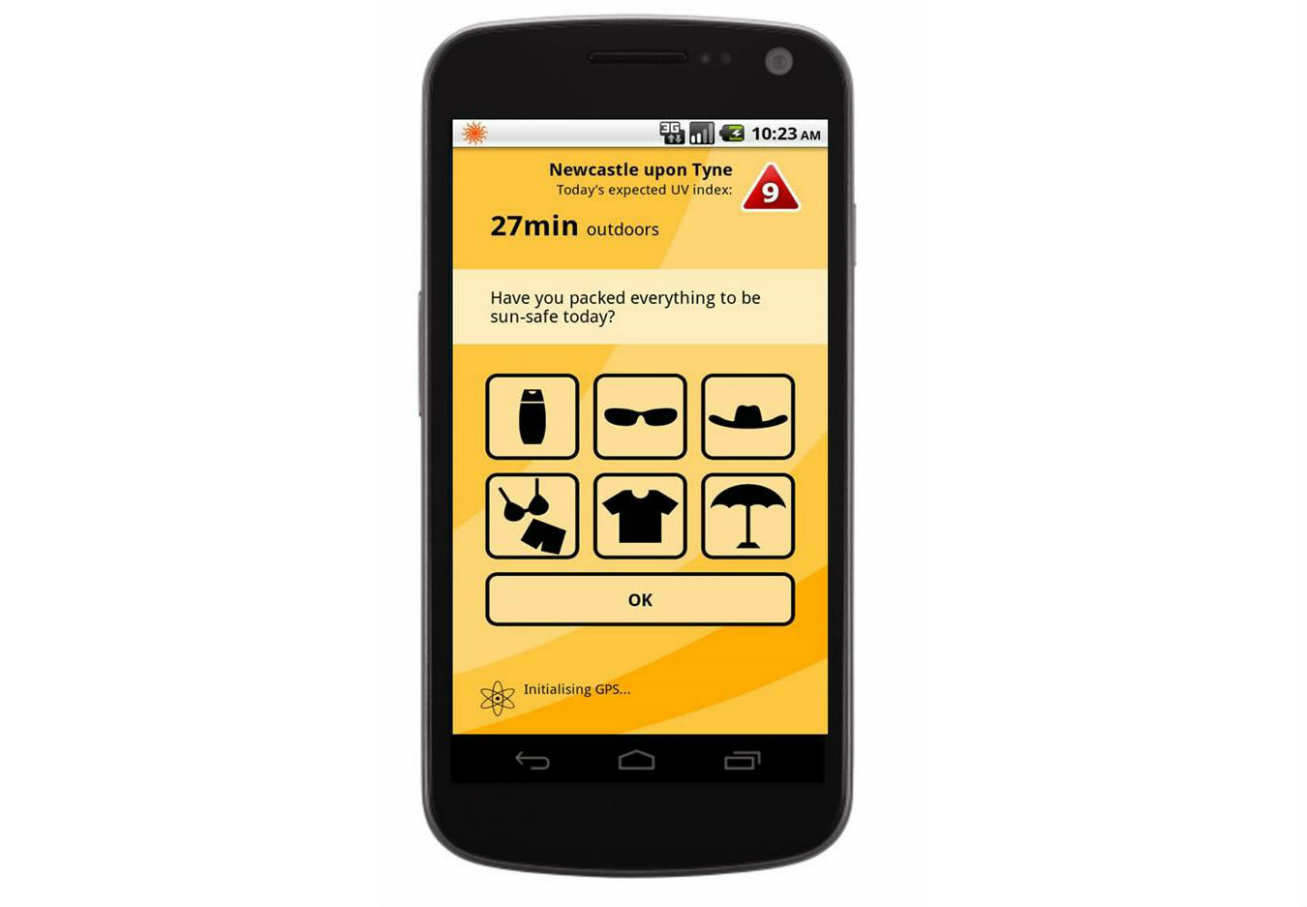
The Sun Alert service main screen.

#### Description of the mISkin App Prototype Mock-Up

The proposed smartphone intervention (*mISkin* app) was developed on the Android platform. The app entails a behavioral intervention using several BCTs to promote sun-protective behaviors amongst holidaymakers. Table 1 describes the main features of the *mISkin* app with explicit justification of inclusion based on evidence.

### Stage 3: Evaluation of the mISkin App Prototype

#### Findings

Table 2 summarizes the feedback provided by participants on the specific features of the *mISkin* app and the suggested changes to improve ease-of-use, appeal, and usefulness.

**Table 2:** Feedback on the *mISkin* app provided by participants and changes introduced.

Intervention component	Category of suggested changes	Sample quotes	Changes implemented in the intervention
Skin assessment	Order of questions	“Information about specific skin types was quite useful.” “Having the question about the skin reaction before the color of the skin in the skin assessment.”	The question order about skin reaction was changed.
Videos	Video content	“It would be quite useful to see the clip again after seeing all the information in the little quiz or having the video after.” “I like the practical advice about how much sunscreen to put on. I would say it would be more effective if it didn’t leap straight into skin cancer and it started with choose a good sunscreen and then link to the consequences of not doing it.” “I think it would be quite good to have a checklist at some point that we could look up.”	A video menu was added to make navigation through different sections easier (eg, how to apply sunscreen and instructions for other sun-protection behaviors)
	Video length	“Instead of having a very long video having the different sections.”	Different snapshots of the videos were added to the menus, reducing the information displayed. The video menu was organized so that skin cancer information is the last video displayed.
Sun safety quiz	Content	“In the quiz, instead of saying just true or false, say something like you’re correct or that’s wrong.” “I like the quiz bit; you can do it once.”	Explicit feedback on performance was added.
	Confusing statements in quiz questions	“Tricky question the one about sunburn doubles the risk of skin cancer.”	The sentence was changed to “increased risk of melanoma.”
Prompts	Content	“Like you say stay out of the sun between 10 and 4pm. Give some ideas how to do that. Like say have a nice long leisurely lunch sounds much better than you must stay in the shade between 10 and 4pm.”	Some suggestions on how to seek shade between 10 AM and 4 PM were added to the reminders.
	Frequency	“I quite like it particularly the prompts. I would probably like to have a bit more, have the opportunity to remind me a bit further.” “I like the idea of a sunscreen reminder app that I could set up to my preference.”	A preference setting was added to the alert service, so that reminders are customizable (ie, 30 minutes to 2 hours).
UV^a^ photographs	Reaction	“It’s quite scary though, is it? I’ve seen a few of these before and it always makes you feel I should put more on.” “It’s a good idea to have it in and it’s better than when that woman talking. Just put it a bit earlier in the app. it’s the shock factor that would make you think: oh I don’t want to look like this. So I suppose it should be in...” “It’s quite scary; it might put me off the app. that the last thing I want to see on holiday.”	UV photographs were moved to the video menu (participants visualization of these depends on their choice) and were placed as the last available option to be seen. A brief explanation about the meaning of the UV photographs was also added so that participants are aware of what it implies and know what to expect.

^a^UV: Ultraviolet.

#### Ease-of-Use of the mISkin App

Overall, the intervention was well-received by participants and described as appealing and interesting to use.

Having the information is good as I don’t think people know. Also the reminders are good as on holidays sometimes you forget and it’s good to be reminded.

Female, 32 years old, with skin type III

I like the tone about you’re on holidays, here is how to be on holiday without “killing yourself,” like the kind of how to enjoy your holiday.

Male, 28 years old, with skin type IV

Most users found that the app was useful and stated that they would use it on their holidays. There was a general satisfaction with the app.

I think I would do (use) and I think especially if you’ve got children as well, you know, I think that’s really good. I mean my children are all grown up now but I do have a year old granddaughter so it’s, I think it would be good because when you’re a busy mum or grandparent and you sometimes forget to do things when there’s a lot going on so...

Female, 55 years old, with skin type II

Users also mentioned the ease-of-use of the app and how the app is “intuitive to interact with” and provides information that is understandable.

Information needs to be there so that people know and can protect themselves. It was simple information and got the message over. I don’t think it was boring, it was informative and that’s something you need.

Female, 55 years old, with skin type II

#### Appeal of the Different Interfaces of the mISkin App

All participants provided positive feedback regarding the appearance of the *mISkin* app, stating that the background image, design, graphics, and color scheme were all appealing. “I quite like the design,” said a female respondent aged 55 years, with skin type II.

### Stage 4: Refinement of the mISkin App

#### Findings

After the amendments and refinements, the research team produced an optimized *mISkin* app reflecting user preferences. The active ingredients of the intervention were kept the same, with the main refinement being in terms of design to improve usability and acceptability. A final fully functional *mISkin* app was produced to be tested in a pilot randomized controlled trial (to be reported elsewhere).

The main elements of the *mISkin* app are providing general information about the consequences of unprotected sun-exposure, addressing appearance-related concerns, providing instructions for sun-protection, providing demonstration (modeling) on how to perform sun-protection behaviors, prompts for effective sun-protection when outside (via smartphone GPS), and feedback on exposure and protective behaviors. The app also includes a skin assessment questionnaire. Participants are prompted daily (a minimum of 2 times per day) by the app. Each day, participants are also prompted to respond, through the app, to brief questions about their sun-protection practices (ecological momentary assessment).

The *mISkin app* (Figures 4 and 5) has four main menus: (1) “My skin,” (2) “How to be sun smart,” (3) “Sun safety quiz,” and (4) “Sun Alert service.”

First, the “ *My skin* ” menu has a skin sensitivity questionnaire with general feedback on skin type. The BCTs used provide information on the consequences of unprotected sun-exposure for each individual according to skin type.

Second, the “ *How to be sun smart* ” menu contains videos on sun-protection recommendations: *“How to apply sunscreen*,” *“Choosing a good sunscreen*,” “ *Other methods of sun-protection*,” “ *Preventing damage,” “Protecting children*,” “ *Others’ use of sunscreen*,” and skin damage information depicted in combination with UV photographs. The BCTs used in the videos provide information on the consequences of behavior in general, provide information on where and when to perform the behaviors, provide instructions on how to perform the behaviors, and demonstrate the behaviors. The BCTs used in the UV photographs provide information on the consequences of behavior in general and on appearance-based fear appeals.

Third, the “ *Sun safety quiz* ” menu has a game with questions about sun-protection and tanning beliefs, with provision for immediate feedback that would give information on general recommendations for sun-protection. The BCTs used provide feedback on performance, provide information on the consequences of behaviors in general, provide information about others’ approval, provide normative information about others’ behavior, and facilitate social comparison.

Fourth, the “ *Sun Alert service* ” menu prompts about sun-protection a minimum of 2 times per day and with the option to customize these prompts in accordance with participants’ wishes (eg, times and frequency). The BCT used was prompt practice.

Self-monitoring: This refers to the assessment of sun-protection practices between 11 AM and 3 PM if the person is outside (detected by the app) at least once a day and can be customized by participants. The BCT used was prompt self-monitoring of the behavior, as well as of the outcome of it (redness and burning).

UV levels are forecast with an indication of the most effective protection behavior. The BCT used was prompt practice.

## Discussion

### Principal Findings

This paper described a systematic and iterative approach to the development of the *mISkin* app aimed at promoting sun-protection during holidays. The sequential approach to development integrates different strands of evidence to inform the design of an evidence-based intervention. A systematic review on previously tested interventions to promote sun-protection provided cues and constraints for the design of this intervention. The development and design of the *mISkin* app also incorporated other sources of information, such as other literature reviews and experts’ knowledge and experience. The developed prototype of the *mISkin* app was evaluated by engaging potential holidaymakers in the refinement and further development of the *mISkin* app through usability (ease-of-use) and acceptability testing of an intervention prototype. All 17 participants were satisfied with the *mISkin* prototype and expressed willingness to use it. Feedback on the app was integrated in the refinement process to produce a final fully functional app before a formal test in a feasibility study. The feasibility and acceptability of the *mISkin* app has been formally tested in a pilot randomized controlled trial (Trial ID: ISRCTN3943558), which will be reported elsewhere.

### Limitations

While the views of the 17 participants were coherent and data saturation was achieved, not all groups of potential users were similarly represented and it is possible that a more extensive engagement of potential users would lead to further improvements in acceptability and usability. The *mISkin* app was only developed for devices using the Android operating system, limiting the possibility of including users owning other smartphones (eg, iPhone and Blackberry). It is also important to highlight that the participants’ views were based on only visualizing a prototype intervention aimed to be delivered during holidays. Views of using the app could change if participants were given the possibility of interacting with the *mISkin* app in a real scenario of holidays. It would have been useful to have a group of users that tried out the app on their own, followed by an interview about their experiences (ie process evaluation built into a pilot acceptability and feasibility study). This would provide further insight into how people perceived and used the app in their own time, which may be different from when a researcher is present [49]. The study did not explore what participants would want to see in an app for sun-protection during their holidays. Instead, they were shown the prototype of the *mISkin* app, potentially losing their general and a priori ideas about what should be in a sun-protection app.

### Comparison With Other Studies

User-centered design is an approach that entails the involvement of potential users in the design process of a product (eg, intervention materials) by tackling their specific needs [22]. This process usually involves eliciting feedback from users by showing a prototype version of the intervention and implementing formative usability testing [22]. Other studies have used formative evaluation and shown similar findings on the potential of a smartphone intervention [24,50].

A key feature of the *mISkin* app is the Sun Alert Service and we were aware of the risk of participants becoming annoyed with receiving alerts or reminders within the *mISkin* app. As in similar studies, participants were keen to gain control over the settings for the Sun Alert service [24,50], in order to be able to customize the alerts (ie, quantity, frequency, and timings). Participants also raised concerns over the information about skin cancer and how this might impact the acceptability of the app and their willingness to use it. Other studies have identified similar challenges when communicating behavior change and health-related awareness [50]. The issue about avoiding provoking adverse emotional reactions becomes more prominent when discussing the UV photographs, as participants had mixed opinions about this feature. However, in the context of sun-protection, the evidence suggests that negative emotional reactions play an important role as predictors of sun protection [51].

### Conclusions

This study summarizes the sequential and iterative process of developing the *mISkin* app, which was aimed at promoting sun-protection. Prototype testing provided useful information regarding users’ views on and experiences from engaging with the *mISkin* app. Suggestions made by participants were incorporated in the refinement and development of a fully functional *mISkin* app. The optimized version of the app is ready for formal testing in a feasibility pilot study, to explore whether it is a feasible vehicle to deliver an intervention aimed at improving sun-protection amongst holidaymakers.

## Appendix

Multimedia Appendix 1 Co-design interview topic guide.

## Abbreviations

BCT: behavior change technique
NHS: National Health Service
UV: ultraviolet
