# Supplementary material for: Systematic and Iterative Development of a Smartphone App to Promote Sun-Protection Among Holidaymakers: Design of a Prototype and Results of Usability and Acceptability Testing
Source: JMIR Res Protoc. 2017 Jun 12;6(6):e112. doi: 10.2196/resprot.7172 (PMC5484792; doi:10.2196/resprot.7172)
Supplement: Multimedia Appendix 1 [file resprot_v6i6e112_app1.pdf]

Multimedia Appendix 1

Co-design interviews topic guide

Introduction

The purpose of this study is to provide feedback on a new mobile phone intervention that could support holidaymakers in protecting their skin.

There are no right or wrong answers to the questions. Take your time to answer each question and, if you prefer, take a few minutes to think about it before answering.

Feedback on the mobile phone intervention app

Procedures: participants will be given a prototype of the mobile phone app on an Android phone and will interact with it for about 5-10 minutes. After this initial procedure participants will be asked about the specific content and graphical aspect of the app.

Opening questions:

Would you anticipate any advantages/disadvantages of a mobile-phone intervention like this, to use during your holiday? (Prompts: usefulness, intrusiveness, holidays' interference).

Feedback prompts for each feature of the app: comprehension, understanding, if information was appealing/interesting, motivation to comply; information specific to your skin type; what things would you do differently or think should be improved?

Final questions:

Do you think this intervention would help you to protect your skin from the sun? If yes, how?

Would you use this app?

If you want to use this app, would this motivate you to take your mobile with you on your holidays and to the beach?
